# Cortisol Variations to Estimate the Physiological Stress Response in Horses at a Traditional Equestrian Event

**DOI:** 10.3390/ani13030396

**Published:** 2023-01-24

**Authors:** Sergi Olvera-Maneu, Annaïs Carbajal, Paula Serres-Corral, Manel López-Béjar

**Affiliations:** 1 Department of Animal Health and Anatomy, Universitat Autònoma de Barcelona, Bellaterra (Cerdanyola del Vallès), 08193 Barcelona, Spain; 2 College of Veterinary Medicine, Western University of Health Sciences, 309 East Second Street, Pomona, CA 91766, USA

**Keywords:** saliva, equid, festivals, stress, hypothalamic–pituitary–adrenal axis

## Abstract

**Simple Summary:**

The centuries-old patronal festivals in Menorca (Spain) represent an alternative to the common use of horses. During festivals, horses are exposed to potential sources of transient stress. This study aimed to evaluate the variations in salivary cortisol concentrations to estimate the physiological stress response in horses at the Menorca patronal festivals. For this purpose, the salivary cortisol variations before, during, and after the celebrations were assessed using an enzyme immunoassay. All the samples collected during festivals were significantly higher than the control group samples (*p* < 0.05). Within twenty-four hours after the end of the celebrations, cortisol concentrations returned to baseline levels and did not differ significantly from the control group (*p* > 0.05). Overall, the study found that the horses’ participation in the festivals resulted in a transitory and measurable stress response.

**Abstract:**

In many countries, horses remain involved in traditional equestrian events such as those celebrated in Menorca (Balearic Islands, Spain) every year since at least the 14th century. The present study aimed to evaluate the variations in salivary cortisol concentrations to estimate the physiological stress response in horses at the Menorca patronal festivals. Two different editions (years 2016 and 2018) of the festivals in honor of the Virgin of Grace in Maó (Menorca, Spain) were studied. Nineteen and seventeen Pure Breed Menorca stallions were included in the study, respectively. The stallions were aged between seven and twelve years. During celebrations, samples were collected before the start of the festivals between 8–9 a.m. and during the festivals at 8–9 p.m. On the second day of celebrations, the samples were collected at 8–9 a.m. and 3–4 p.m. Finally, on the day after the festivals, one sample was collected at 8–9 p.m. Additionally, a control group was sampled at 8–9 a.m., 3–4 p.m., and 8–9 p.m. Salivary cortisol concentrations were assessed by using a commercial enzyme immunoassay kit specially validated to quantify salivary cortisol in horses. Salivary cortisol concentrations did not show significant differences between sampling hours in the control group (*p* > 0.05). All the samples collected during festivals were significantly higher than samples of the control group (*p* < 0.05). Within the twenty-four hours after the end of the celebrations, cortisol concentrations returned to baseline levels and did not differ significantly from the control group (*p* > 0.05). Hence, the present study describes that the participation of the horses in these particular acts generate an acute and transitory stress response. Overall, the current work provides a reasonable basis for future research on the stress physiology and well-being of horses participating in traditional celebrations or similar events.

## 1. Introduction

In many countries, particularly in the Mediterranean region, equestrian celebrations, games, and tournaments originating from religious and historical events represent an alternative to the common use of horses [1]. In Menorca (Balearic Islands, Spain), every summer, cavalry events have been held in honor of the town’s patron saints since at least the XIV century. These events are popularly known as patronal festivals or celebrations, and their origin remains to be clarified. However, the principal accepted hypothesis dates their origin during the Medieval period and is strongly related to the creation of the commissions in charge of the churches. Those commissions had the purpose of going around the town, riding horses, and collecting money to upkeep the town’s ecclesiastic building and celebrate acts in honor of their patron saint. At present, the main characters of the celebrations are the horses, and the most commonly used breed is the Menorca Purebred horse. This native breed is excellent for riding and performing Classical and Menorca dressage, a distinctive type of dressage in which the horse practices exercises and movements similar to those performed during the patronal festivals [2]. The centuries-old celebrations have helped to maintain and improve this endangered native horse breed, and currently, there is a census of approximately 3700 individuals, mainly located in Menorca. In the last decade, the social interest in the welfare of the participant horses has grown substantially, since the animals must face potentially stressful situations during the acts. Hence, evaluating their stress response could be the primary approach to success in the horses’ performance and well-being, as described in other common equestrian disciplines [3]. As well documented in athletic animals such as horses, following physical exercise, specific changes in metabolic reactions occur in athletes leading to several changes in the body, mainly in the circulatory, respiratory, endocrine, and neuromuscular systems. Changes taking place in these systems simultaneously and in an integrated manner are aimed at maintaining homeostasis in the body [4,5].

The stress response evaluation in horses can be performed by assessing behavioral and/or physiological indicators [6]. Cortisol, the final product released into the blood after activating the hypothalamic–pituitary–adrenal axis (HPA), has become one of the most used physiological indicators to measure the stress response, not only in horses but also in other species [7,8,9]. Different studies have reported a positive correlation between cortisol concentrations and heart rate, respiratory rate, rectal temperature [10], eye temperature [11,12], or blood lactic acid [13], all of them indicators of the intensity and effort of the exercise, and therefore indicators of the physiological stress response in horses. Cortisol secretion is characterized by a circadian rhythm that peaks in the early morning, with the nadir phase occurring in the evening [14,15]. Furthermore, cortisol has many supportive and advantageous physiological roles, including maintaining and restoring body homeostasis [8,9]. Hence, cortisol participates in the tolerance and adaptation of the horse to short-term exercise demands [3,9].

The measurement of blood cortisol in horses has long been the most common method for measuring the HPA axis activity. However, salivary cortisol analysis has recently become increasingly popular to assess the adrenocortical response [7,16,17]. Salivary cortisol represents the biologically active form of the hormone [7,18], and a strong association between levels in blood and saliva has been described in horses. After an adrenocorticotropic hormonal challenge in horses, Peeters et al. [16] described that total blood cortisol concentrations might account for 80% of salivary cortisol concentrations and vice versa. Additionally, the salivary cortisol measurement offers a non-invasive and easy tool to perform a repeated and “non-disturbing” sampling for the animal [19,20], ideal in cases where blood collection may be difficult or impossible. Furthermore, compared to blood sampling, saliva sampling does not require trained and qualified personnel to collect the samples [19,20].

The stress response in horses exposed to different exercises and sports activities such as competition [21,22,23], recreational activities [24,25,26], and training [27,28,29] has been well studied, including the measurement of salivary cortisol concentrations. To date, no studies have been performed to investigate the physiological stress response of the horses at the Menorca patronal festivals. Interestingly, Pazzola et al. [1] explored the stress-related physiological changes in horses of the “Sa Sartiglia” tournament (Sardinia, Italy), also celebrated since centuries ago and with some similarities with the Menorca festivals.

The present study aimed to evaluate the variations in salivary cortisol concentrations to estimate the stress response in stallions at the Menorca patronal festivals. We hypothesized that the participation of the horses in the celebrations would produce an acute and transitory stress response that would be reflected by an increase in the salivary cortisol concentrations during the celebration days.

## 2. Materials and Methods

### 2.1. Celebrations

**Figure 1:**
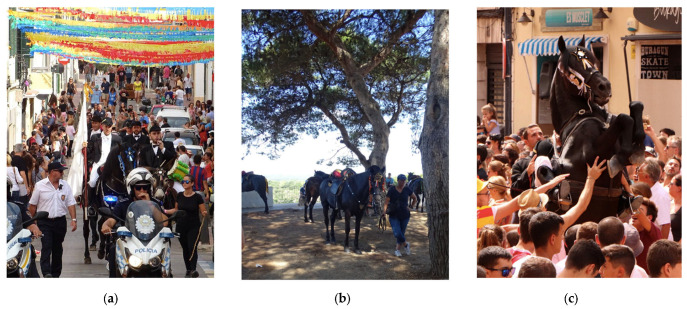
(**a**) Horses and riders during the parade to the church. Photo credits by Trevor Fryatt. (**b**) Horses resting during the celebration of the mass. Photo credits by Trevor Fryatt. (**c**) Horse performing the “bot” in the middle of the crowded main square during a jaleo round. Photo credits by Catalina Pasqual.

Every year on 7 and 8 September , more than a hundred stallions and their riders participate in the patronal festivals in honor of the Virgin of Grace in Maó (Menorca, Spain) in the presence of thousands of people. The festivals are celebrated once a year and include different religious and popular events. Both days of the celebrations follow a similar structure. The horses are transported into the event site by trailer or walking, depending on their proximity. The festivals begin with the horses’ parade to the church (Figure 1a), where religious events are celebrated. When the masses are held, the horses rest for approximately 1.5–2.5 h (Figure 1b), depending on the day of the festival. Shaded areas and ad libitum running water are available for the horses during the resting periods. Once the religious acts are ended, the horses head towards the village’s main square, where the most popular event, named “jaleo”, is celebrated (Figure 1c). All the riders and horses pass through the main square in groups of five and make the horses rear up several times for a few seconds (Figure 1c), performing the “bot” (walking courbette). The horses enter the square three times (per day), and this process is popularly known as “jaleo round”. As established by the authorities, each horse during the jaleo round must remain less than 1.5 min in the square. Between jaleo rounds, horses rest for approximately 1–1.5 h.

### 2.2. Animals, Sampling, and Ethics

The design of the study was planned according to the different phases and necessities of the patronal festivals. Two different years of the festivals were studied (2016 and 2018, named A and B, respectively). Nineteen and seventeen Pure Breed Menorca stallions aged between seven and twelve years were included in the study for the A and B editions, respectively. Animals included in the study were regular participants in the festivals. All individuals were healthy and lacked any history of illness during the studied period. The body condition score (BCS) was evaluated using the 0 (emaciated) to 5 (extremely fat) BCS scale [30]. For all the stallions evaluated, the BCS was 3 (moderate to good body condition).

**Figure 2:**
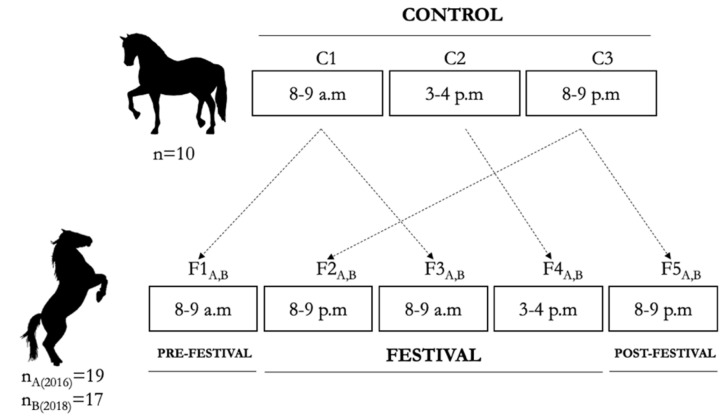
Schematical representation of the sampling timeline. Control samples were taken between 8 and 9 a.m. (C1), 3 and 4 p.m. (C2), and between 8 and 9 p.m. (C3). During patronal festivals samples were taken the morning of 7 September, before the start of the festivals, between 8 and 9 a.m. (F1), and during the festivals between 8 and 9 p.m. (F2). On 8 September, samples were taken between 8 and 9 a.m. (F3) and 3 and 4 p.m. (F4). Finally, the last sample was collected the day after the festivals, on 9 September, between 8 and 9 p.m. (F5). The same sampling scheme was followed in both studied editions (A and B).

Saliva was collected using sterile mounted swabs (Sugi^®^, Eschenburg, Germany), specially designed to absorb secretions, rubbing the cheek mucosa and under the tongue for approximately twenty seconds. Saliva was recovered from the swab, placing it into a sterile syringe, and pressing the plunger until the obtention of the maximum possible volume. Samples were stored under freezing at −20 °C and processed and analyzed within six months from collection. We followed the timeline presented in Figure 2. The first sample was taken on the morning of 7 September, between 8–9 a.m. (F1), before the start of the festival. The following sample was collected between 8–9 p.m. (F2). On 8 September, the samples were taken between 8–9 a.m. (F3) and between 3–4 p.m. (F4). Finally, the last sample was collected the day after the festival, on 9 September, between 8–9 p.m. (F5). The same protocol and timeline were followed in both studied editions. The horses stayed at their stables when the samples were collected before and after the celebrations (F1 and F5). In contrast, during the patronal festivals (F2, F3, and F4), the samples were collected in situ, trying to avoid the disturbance of the events.

Additionally, a control group of ten stallions, regular participants at the patronal festivals, was sampled to set the salivary cortisol baseline values. The sampling days in the control group were kept apart from any housing- and handling-related changes or disturbances. All the stallions included in the control group were healthy and lacked any history of illness during the studied period. The body condition score (BCS) was evaluated using the 0 (emaciated) to 5 (extremely fat) BCS scale [30]. For all the stallions evaluated, the BCS was also 3 (moderate to good body condition). From the control group, two replicates (one in August and another in September of 2019) were collected to avoid potential intra-seasonal influence on salivary cortisol concentrations [15]. Samples were collected between 8–9 a.m. (C1), 3–4 p.m. (C2), and 8–9 p.m. (C3). The followed sampling procedure was the same as the abovementioned. Sample C1 was used as a control for samples F1 and F3, C2 was used as a control for sample F4, and C3 was used as a control for samples F2 and F5 (Figure 2).

During the study, all animals were managed following the principles and guidelines of the Ethics Committee on Animal and Human Experimentation from the Universitat Autònoma de Barcelona (Barcelona, Spain) and following the Directive 2010/63/EU on the protection of animals used for scientific purposes. No other manipulation different from the salivary collection was performed. Additionally, informed consent from the owners of the horses was obtained before the start of the study.

### 2.3. Cortisol Quantification and Biochemical Validation of the Enzyme Immunoassay

For horses’ salivary cortisol analysis, a commercial Enzyme-Linked ImmunoSorbent Assay (ELISA) kit (Neogen Corporation©, Ayr, UK) was used. According to the manufacturer, cross-reactivity of the ELISA cortisol antibody with other steroids was prednisolone 47.4%, cortisone 15.7%, 11-deoxycortisol 15.0%, prednisone 7.83%, corticosterone 4.81%, 6β-hydroxycortisol 1.37%, 17-hydroxyprogesterone 1.36%, and deoxycorticosterone 0.94%. Steroids with a cross-reactivity <0.06% are not presented. Salivary samples were analyzed at 1:1 dilution and the optical density was read using a microplate reader (Sunrise^TM^ basic microplate reader, Tecan Austria GmbH, Grödig/Salzburg, Austria) at 450 nm.

The ELISA kit was biochemically validated for horses and saliva by following the criteria of precision, specificity, accuracy and sensitivity [31]. The validation tests were performed using a constituted pool created with 30 µL from each of the salivary samples included in the study. The precision of the test was assessed by calculating the intra-assay and inter-assay coefficients of variation from all the duplicated samples. The specificity of the test was evaluated by calculating the linearity of the dilution using 1:1, 1:2, 1:5, and 1:10 dilutions of the salivary pool diluted with the ELISA buffer provided by the kit. The accuracy was assessed through the spike-and-recovery test, calculated by adding to 100, 75, and 25 μL of the salivary pool volumes of 25, 75, and 100 μL of three different cortisol concentrations provided by the ELISA kit (0.2, 0.4, and 1 ng/mL). Finally, the sensitivity was given by the smallest cortisol concentration that the assay could detect and measure. For the present study, the intra-assay and inter-assay coefficients of variation were 6.2% and 12.2%, respectively. In the dilution test, the obtained and expected cortisol concentrations for saliva were significantly correlated (r = 0.99, *p* < 0.01). In the spike-and-recovery test, the average of recovery percentage was 107.6 ± 10.0%, and the obtained and theoretical concentrations were significantly correlated (r = 0.99, *p* < 0.01). The assay’s sensitivity for salivary cortisol was 0.07 nmol/L. The biochemical validation of the assay showed reliable results and demonstrated the precision, specificity, accuracy, and sensitivity of the assay in measuring horse salivary cortisol.

### 2.4. Statistical Analysis

Data were analyzed using R Studio software (R version 3.4.4) and graphically represented using Graph Pad Software Inc. (GraphPad Prism, version 8.0.2; Graph Pad Software Inc., San Diego, CA, USA). The significance level in all data was set at *p* < 0.05. For the data obtained in the dilution test and the spike and recovery test of the ELISA kit biochemical validation, a Pearson’s Product Moment correlation was applied between the expected and obtained values. The normality of the data was evaluated using a Shapiro–Wilk test and salivary cortisol concentrations were log transformed to achieve the normal distribution (*p* > 0.05). To analyze the effect of the month and the sampling hour in the salivary cortisol concentrations of the control group, a Linear Mixed Effect model was used considering the sampling day (D1 or D2) and the sampling hour (samples C1, C2, and C3) as fixed factors and the individuals as random factors followed by an ANOVA performed on the fitted model. To evaluate the changes in salivary cortisol concentrations during the festivals a Linear Mixed Effect model was used, setting the edition of the celebration (A and B) and the sampling hour (samples F1_A,B_, F2_A,B_, F3_A,B_, F4_A,B_, and F5_A,B_) as fixed factors and the individuals as random factors. An ANOVA was performed on the fitted model. Finally, the Dunnett’s post hoc test was performed between the control and the patronal festival group. Hence, sample C1 was compared with F1_A,B_ and F3_A,B_, C2 was compared with F4_A,B_, and finally, C3 was compared with F2_A,B_ and F5_A,B_.

## 3. Results

### Salivary Cortisol Concentrations

**Figure 3:**
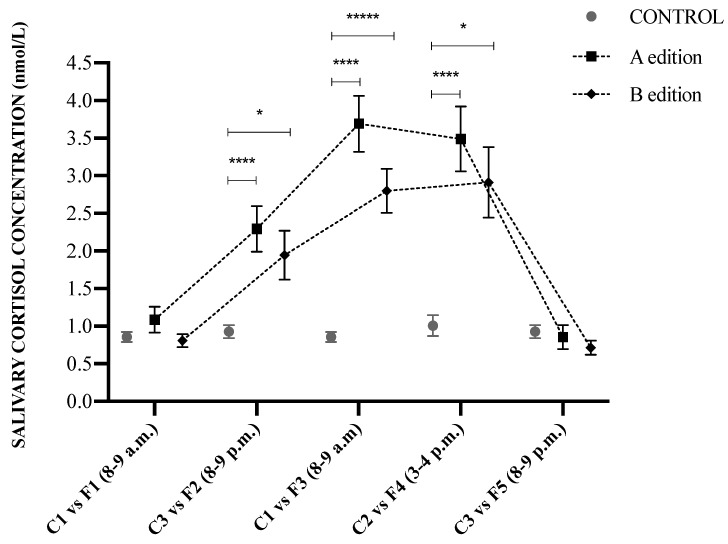
Mean ± SEM salivary cortisol concentrations (nmol/L) from the evaluated horses during the festival days (Fx) and the control group (Cx). Upper asterisks indicate significant differences between obtained salivary cortisol concentrations.

Changes in salivary cortisol concentrations are presented in Figure 3. Salivary cortisol concentrations were not influenced by either the sampling day or the sampling hour (*p* > 0.05) in the control group. A significant effect of the sampling hour (*p* < 0.05) on salivary cortisol concentrations was detected in the patronal festival group, while no influence of the edition (A and B) was stated (*p* > 0.05). The Dunnett’s post hoc test showed that samples collected in F1 (8–9 a.m.) were not statistically significant in comparison to C1 samples (8–9 a.m.) (*p* > 0.05). The first sample collected at the festivals, F2 (8–9 p.m.), was significantly different from the control group C3 (8–9 p.m.) in both editions of the study, A (*p* < 0.001) and B (*p* < 0.05). Salivary cortisol concentrations were higher in F3 (8–9 a.m.) (*p* < 0.001) compared to the control group C1 (8–9 a.m.). Sample F4 differed significantly from C2 in A (*p* < 0.001) and B (*p* < 0.05). Finally, within the twenty-four hours after the end of the celebrations, F5 (8–9 p.m.) samples were not significantly different (*p* > 0.05) compared to the samples obtained from the control group C3 (8–9 p.m.).

## 4. Discussion

The aim of the present study was to evaluate the physiological stress response in horses at the Menorca traditional equestrian celebrations by measuring the salivary cortisol variations. The authors hypothesized that horse’s participation results in an acute and transitory stress response reflected in an increase in the salivary cortisol values during the festivals. The results corroborated the authors’ hypothesis. For the first time, the generated physiological stress response in these stallions was described. Furthermore, the present results showed that cortisol levels returned to baseline within the first twenty-four hours, suggesting the participation of the horses in these festivals as an acute and transitory stressor.

To set the salivary cortisol baseline levels, a control group of ten stallions was sampled at three different hours (8–9 a.m. (C1), 3–4 p.m. (C2), and 8–9 p.m. (C3)), repeating the process in two different days (once in August and another in September of 2019). This was performed to avoid potential sources of intra-seasonal variations [32,33,34]. Baseline salivary cortisol concentrations detected in the present study showed similar values to other jumping and dressage horses [20,35]. Additionally, salivary cortisol concentrations of the control group were not significantly influenced by the sampling day or the sampling hour. Thus, results suggested the absence of circadian rhythm in the evaluated horses, contrary to what was reported in other investigations [14,36]. Different authors have suggested that changes in handling, habitat, generalized disease, and intensive exercise can disturb the cortisol circadian secretion pattern in horses [15,37,38]. All abovementioned disturbing factors can be discarded in this study, since during the sampling day, the horses of the control group were kept apart from any housing- and handling-related changes or disturbances, and they were clinically healthy during the studied period. Hence, the management conditions may not have driven the absence of circadian rhythm. Decreasing the interval time between sample collection could help to accurately measure the presence of a possible circadian rhythm in these stallions [14,33]. Further research is needed to clarify these findings.

Various studies have shown how horses anticipate a potential stressful event such as loading transport vehicles [39], race competitions in comparison to regular training [40], or trekking courses [41]. In the present study, the morning sample obtained before the start of the festivals (F1_A,B_) did not differ significantly from sample C1. This finding suggests no anticipatory stress response, partially explained because the horses remained in their stables while the samples were taken.

In exercised horses, salivary cortisol has been demonstrated to be a valuable indicator of stress levels [20,21,42]. In the present study, salivary cortisol concentrations obtained during festival days (F2_A,B_, F3_A,B_, and F4_A,B_) were significantly higher than those obtained from the control group (C1, C3, and C2). The observed increase is intended to compensate for the effects of the physical and environmental stressors to which the horses were exposed during those days (e.g., activity increase, crowded places, change in their habitual habitat, presence of other stallions, transport from their origin place to the town or music, among others). The results also showed that cortisol concentrations increased in parallel to the duration of the festivals, suggesting that that cortisol’s increase was related to the duration and intensity of the exercise [9,42]. Compared to the control group, salivary cortisol increased from ~100% on the first day of the celebrations to ~300% (edition A) and ~200% (edition B) on the second day’s morning. In addition, a similar physiological stress response to the festivals was found in both studied editions. The observed variations during the festivals can be compared to the results obtained by Kedzierski et al. [27], which showed an increase of ~150% immediately after a short exercise of moderate intensity (800 m galloped distance) in Arabian horses. Furthermore, Peeters et al. [22] showed that salivary cortisol was ~340% higher after a cross-country road trip than just before the start. Other studies have reported higher increases in cortisol concentrations than those observed in the present study, as for example the results reported by Schmidt et al. [39], who documented a salivary cortisol increase higher than 600% during road transport. Based on these studies, it is likely that both high- and low-intensity exercise (long or short) induce a cortisol increase. However, most studies have yet to have an exact measure of exercise intensity or a standardized time and frequency of post-exercise cortisol measurements. Overall, the results of the present study suggest that the stress response generated during the festivals was not exceeding the stress response generated in other activities to which domestic horses are regularly exposed.

Throughout the year, the evaluated horses are trained to be in their best physical condition to ensure a successful performance and human safety during the celebration days. Trainings include practicing the typical gaits and movements performed during the festivals. The results of the present study are aligned with results obtained in our preliminary study (unpublished data), in which the stress response of horses during a festivals’ training session was evaluated. Salivary cortisol concentrations increased ~240% after one hour of training. Therefore, the regular training of these horses would allow them to properly prepare for the festivals’ demands.

Different investigations have suggested that cortisol, the main glucocorticoid in horses, plays an essential role in metabolism and energy balance [43]. In medium–short-term action, cortisol acts as a catabolic hormone, increasing the energy production and restoring horses’ homeostasis during and after exercise [9,44,45]. Hence, exercise’s homeostasis disturbance would be compensated by activating the HPA axis, increasing circulating cortisol, among other hormones [9]. The present study showed that salivary cortisol concentrations were highly variable between individuals during the celebrations. This result follows other reported results of horses exposed to different types of exercise and conditions (e.g., [1,16,22,23,25]). The interindividual variability detected in salivary cortisol concentrations could be partially explained by the genetic conditioning of the horses [27], the interaction with their rider [23,46], or the experience of the animals participating in the festivals.

Sample F5 (24 h post-festivals) did not show significant differences compared to sample C3 of the control group. Thus, cortisol returned to baseline levels within the first twenty-four hours following the event. The return to salivary cortisol baseline levels has been reported to be different depending on the stimulus the horse has been exposed to. For example, after an exogenous adrenocorticotropic stimulation, salivary cortisol concentrations returned to baseline levels after 180 min [16], between 30 min and 1 h after a jumping course [20,47], or 2 h after unloading from a trailer, irrespective of the transport’s duration [48]. On the other hand, we observed in our preliminary study (unpublished data) that horses returned to salivary cortisol baseline levels after a training session to participate in the festivals within two hours after finishing the exercise. In addition, in line with the present study’s findings, Pazzola et al. [1] reported the return to cortisol baseline values within the day after the end of the traditional tournament of “Sa Sartiglia” (Oristano, Italy). Altogether, the return to basal levels within 24 h after the end of the event suggests the restoration of the homeostatic balance and therefore the absence of a chronic stress situation produced by the participation of these horses in the festivals.

## 5. Conclusions

Overall, the current study provides a basis for future research on the stress physiology of horses participating in traditional celebrations or similar events. For the first time, this study describes that the participation of the horses at the Menorca patronal festivals generates an acute stress response reflected by a transitory salivary cortisol increase. The results of the present study show how salivary cortisol concentrations increased progressively, reaching the maximal concentrations on the second day of the festivals, and returning to the baseline levels within the day after the acts. The authors encourage future studies to investigate the possible long-term consequences on the health and welfare of the horses participating in these events.

## Data Availability

Data presented in this paper have not been published or stored elsewhere, but are available on request from S.O.-M.
